# The effect of timely initiation of complementary feeding and vitamin A supplementation on acute malnutrition among children aged 6–59 months attending Hamusit Health Centre, Northwest Ethiopia, 2021: A cross-sectional study

**DOI:** 10.1016/j.heliyon.2021.e08449

**Published:** 2021-11-22

**Authors:** Ermias Sisay Chanie, Zeleke Dagne, Melkamu Senbeta Jimma, Tahir Eyayu, Samuel Nebiyu, Endalk Birrie Wondifraw, Fisha Alebel GebreEyesus, Getaneh Atikilt Yemata, Abenezer Melkie

**Affiliations:** a Debre Tabor University, Debre Tabor, Ethiopia; b Asossa University, Assosa, Ethiopia; c Wollo University, Wollo, Ethiopia; d Wolkite University, Wolkite, Ethiopia

**Keywords:** Acute malnutrition, Children aged 6–59 months, Cross-sectional study, Ethiopia

## Abstract

**Introduction:**

Acute malnutrition is a nutritional deficiency that results either from inadequate energy or protein intake. It is more prevalent in low- and middle-income countries. Even though efforts have been carried out at the global and national level, the burden is still intolerable and it attracts the attention of the government and researchers. Hence, this study aims to assess the magnitude of acute malnutrition and its associated factors among under-five children who attended Hamusit Health Centre from 1st September to 30th January 2021.

**Materials and methods:**

This institution-based cross-sectional study was conducted from 1st September to 30th January on 404 randomly selected under-five children who visited the health centre. Samples were selected using a simple random sampling technique, and the data were obtained using a pre-tested standardized questionnaire. For data entry and analysis, Epi-info 7 and SPSS 21 applications were used, respectively. Bivariable and multivariable binary logistic regression were used to identify associated factors at a 95% confidence interval. Significance was considered at p-value<0.05.

**Results:**

The present research involved a total of 404 children aged 6–59 months. The magnitude of acute malnutrition in this study was 14.4%. Children aged 6–23 months [AOR: 2.92; 95%CI (1.46, 5.85)], vitamin A supplementation [AOR: 0.49; 95%CI (0.25, 0.95)], not timely initiation of complementary feeding [AOR: 2.02; 95%CI (1.06, 3.82)] and children with diarrhea prior to two weeks of the survey [AOR: 2.47; 95% CI (1.28, 4.87)] were significantly associated with acute malnutrition.

**Conclusion:**

A significant number of children aged 6–59 months were affected by acute malnutrition. Younger children, vitamin A supplementation, not timely initiation of complementary feeding, and children with diarrhoea were other factors associated with acute malnutrition.

## 1 Introduction

Acute malnutrition (AM) is a major public health challenge [1], which affects more than 50 million children, and it is particularly serious in children younger than the age of 5 causing short-term case fatality rate and important long-term sequelae [2, 3]. Globally, children with moderate and severe acute malnutrition are approximately 60 million and 13 million, respectively [4].

The effects of malnutrition range from disease potentiating effect to death facilitation leading to 13%–66% mortality in developing countries [5]. Acute malnutrition-associated death is mainly due to diarrhoea in those admitted children with severe malnutrition as reported in Zambia [6], and diarrhoea was found to be the determinant factor of malnutrition in Ethiopian community-based studies [7, 8, 9]. World Health Organization (WHO-2012) revealed that 60% of the death was associated with malnutrition among under-five children who were admitted in hospitals [10]. Other disease conditions such as malaria were also predictors of malnutrition [11]. Ethiopian Demographic and Health Survey, 2016 (EDHS-16) reported around 24% of children to be underweight from community survey [12]. Another study in Burkina Faso identified child sex, birth weight, child comorbidity, and maternal education as determinant factors of malnutrition [13]. Several studies revealed that caregivers who washed their hands regularly were less likely to have acutely malnourished children [14, 15, 16]. Safe child faeces disposal practice had been a preventive behaviour regarding malnutrition among children aged 6–59 months as observed from previous studies [14, 16]. Other comorbidities such as HIV/AIDS, diarrhoea, various chronic diseases were identified as factors aggravating malnutrition status in many earlier studies. For example, children with diarrheal disease were more malnourished than those with no diarrhoea [17, 18, 19, 20], as diarrheal disease disrupted luminal mucosal function and resulted in metabolic dysfunction, malabsorption, and nutrient loss which impaired growth, development, and nutritional status [21, 22, 23, 24]. Other chronic and infectious diseases were also factors associated with malnutrition.

Severe acute malnutrition is one of the most important public health problems which is more common in resource-limited countries. Children are highly affected by acute malnutrition more than any other segment of the population. It has a wide range of impact spanning from weak immunity to infectious disease and to poor psychological and cognitive development. Even though efforts at the global and national level have been done, the burden is still intolerable and it attracts the attention of the government and researchers.

There is limited evidence regarding acute malnutrition and associated factors, especially in this particular study area. The finding from this study will serve as a baseline for future research, to augment policy decisions for policymakers, and to government and non-government organizations that are interested in childhood acute malnutrition. Hence, this study aims to assess the prevalence of acute malnutrition and its associated factors among children aged 6–59 months attending Hamusit Health Centre, Northwest Ethiopia.

## 2 Materials and methods

### 2.1 Study design, period, and setting

This institutional-based, quantitative cross-sectional study was carried out from 1st September to 30th January 2021 among children aged 6–59 months. This study was conducted in Hamusit Health Centre. The health centre is located in Hamusit town, a small town located 35-km far from Bahirdar, the capital of the Amhara region. The health centre had 57 staff members (48 professional staff members and 19 administrative staff members) and 12-bed capacities. It serves around 55,426 population in the catchment area and provides both inpatient and outpatient services including paediatrics, minor surgery, maternal and child health, emergency, chronic, youth-friendly service, expanded immunization service, laboratory, and pharmacy. According to the health centre data, more than 12,000 malnourished children visited the health centre in 2020 [25].

### 2.2 Study population

The study population were all randomly selected in the total number of under five children who visited the health centre from 1st September to 30th January 2021.

### 2.3 Inclusion and exclusion criteria

#### 2.3.1 Inclusion criteria

All children aged 6–59 months who visited the health centre for different health conditions, whose mothers/caretakers voluntarily participated in the study were included.

#### 2.3.2 Exclusion criteria

Children whose mother's/care takers refused to participate, and children who visited the health centre more than once with a chance of second-time selection in the study period were excluded.

### 2.4 Sample size determination

The sample size was determined using single population proportion formula [26] with assumptions; p = 50% (institutional-based, cross-sectional study was not previously carried out in the study area to determine the prevalence of acute malnutrition among under-five children visiting the health centre), 95% confidence interval, 5% margin of error (d), and 5% oversampling to account for any unpredictable events.

$$n=\frac{{\left(z_{a/2}\right)}^{2}\times p\left(1-p\right)}{d^{2}}n=\frac{{\left(1.96\right)}^{2}\times 0.50\left(1-0.50\right)}{0.05^{2}}=384$$

Therefore, the final sample size after adding 5% was 404.

### 2.5 Sampling technique and procedure

A simple random sampling technique was used for the selection of the study subjects and the caretakers, preferably mothers of children aged 6–59 months, The s' registration logbook of under-five outpatient diagnosis (U5OPD) used as a sampling frame to select the study participants (i.e., children aged 6–59 months) via lottery method.

### 2.6 Measurement of variables

**Acute malnutrition:** The primary outcome variable of this study was determined by a simple anthropometric index: the so-called mid-upper arm circumference (MUAC). The nutritional status of children was taken as acute malnutrition if the MUAC value was lower than 125- millimetres [27].

**Diarrhoea:** is defined as having three or more loose or watery stools in 24-h, before 2 weeks of the survey [28, 29].

**Timely initiation complementary feeding**-it is time of incitation of additional supplementary food for young child at six months [14, 16].

**ARI (Acute respiratory infection):** Presence or absence of ARI on the study population was determined by the health professionals as the presence of any one or combination of symptoms and signs like cough, sore throat, rapid breathing, noisy breathing, chest indrawing, at any time in the previous 2 weeks [30].

**AFI:** Acute febrile illness is defined as the condition in which a patient has a fever of 38 °C or higher at presentation or history of fever that persisted for 2–7 days with no localizing source [31].

### 2.7 Data collection procedure

After preliminary review, a data extraction tool was used to extract the data from patient medical recording charts and some data were obtained from mothers of children aged 6–59 months. The tool consisted of sociodemographic characteristics of children, maternal-related factors, and other factors that can directly or indirectly influence the nutritional status of children.

### 2.8 Data quality control/management

Before the actual data collection process, a pre-test was conducted on 21 children aged 6–59 months (5% of the total sample size) who visited the Worata Health Center. The data collection tool had to be prepared in English, then translated to Amharic language, and again back-translated to English to ensure consistency and sentence appropriateness and to for a better understanding. One BSc in Nutrition professional and three Diploma in Clinical Nursing professionals were recruited to extract variables from the patient medical records and to collect some important information from mothers of children aged 6–59 months after a face-to-face interview. One day training on the objective of the study, data extraction, data collection technique, and ethical issues was given for data collectors. Data completeness and consistency were checked by the primary investigator every day and necessary corrections were undertaken.

### 2.9 Data processing and analysis

The data first entered in Epi-info 7 were exported into IBM Statistical Package for Social Science (SPSS) Statistics 21 to perform descriptive statistics, cross-tabulation, and to analyse the factors associated with malnutrition. Descriptive statistics were used to express frequency, mean, and percentages. Both Crude Odds Ratio (COR) and Adjusted Odds Ratio (AOR) with 95% confidence interval (CI) were computed to test the strength of association. Binary logistic regression was performed to test the association between independent variables and malnutrition. Variables with p ≤ 0.20 during bivariable analysis were selected for multivariable logistic regression analysis. Factors with p < 0.05 in the final model were declared as significantly associated with acute malnutrition. Hosmer–Lemeshow goodness-of-fit test was used to check model fitness at p > 0.05.

**Ethical approval and consent to participate:** Ethical clearance was obtained from the ethical review committee of the Department of Nursing, College of Health Sciences, Debre Tabor University. A supportive letter was obtained from the Dera Woreda Health office. The verbal informed consent was obtained from the children's mothers or caregivers in the study before data collection. Moreover, children with severe malnutrition were treated, and those with severe complicated malnutrition were admitted/referred.

## 3 Results

### 3.1 Sociodemographic characteristics

A total of 404 children aged 6–59 months with a response rate of 100% who visited Hamusit Health Centre from 1st September to 30th January were included in the present study. The mean age of study subjects with a standard deviation was 26.4 ± 14.6 months. About 212 (52.5%) were male and the majority (83.4%) lived in rural areas. Most of the children (94.3%) lived with both parents. Three out of four 6–59 months old children (75.7%) took vitamin A supplements. For half (51.7%) of the children, the drinking water was from improved sources. From a total of study participants, 53.7%, 39.1%, and 25.2% of children had contracted an acute febrile illness, diarrhoea, and acute respiratory infection, respectively (Table 1).

**Table 1:** Sociodemographic, behavioural and health-related characteristics among 6–59 months old children visiting Hamusit Health Center from 1st September to 30th January 2021 (n = 404).

Variable	Category	Frequency (n)	Per cent (%)
Sex	Male	212	52.5
	Female	192	47.5
Residence	Urban	67	16.6
	Rural	337	83.4
Age variation (months)	6–23	192	47.5
	24–59	212	52.5
Maternal age (years)	16–25	113	28.0
	26–30	129	31.9
	31–34	65	16.1
	35–46	97	24.0
Maternal education	Illiterate	270	66.8
	Literate	134	33.2
Family size	<5	207	51.2
	≥5	197	48.8
Monthly income (n = 400)	150–1600	104	26
	1601–2000	134	33.5
	2001–2500	65	16.3
	>2500	97	24.3
Child living arrangement	With both parents	381	94.3
	With only one parent	23	5.7
The child took Vit. A	Yes	306	75.7
	No	98	24.3
Separate food for the child	Yes	187	46.3
	No	217	53.7
Latrine availability	Yes	170	42.1
	No	234	57.9
Disposal of child faeces into the latrine	Yes	155	38.4
	No	249	61.6
Housing arrangement	Animal and person separate	286	70.8
	Animal and person not separated	118	29.2
Water source	Improved	209	51.7
	Unimproved	195	48.3
Health-related variable	Diarrhoea	158	39.1
	ARI	102	25.2
	AFI	217	53.7
	Malaria (n = 217)	60	27.6

### 3.2 Prevalence of acute malnutrition

The prevalence of acute malnutrition among children aged 6–59 months in Hamusit Health Centre from 1st September to 30th January was 14.4% with 95% CI (11.1%, 17.6%).

### 3.3 Factors associated with acute malnutrition

Binary logistic regression was under-taken at two stages. In the first step, each potential variable was tested for the final model screening and 12 variables (sex of a child, age variation of the child, family size, residence, vitamin A supplementation, latrine availability, living arrangement (whether the room for animals and people were separate), drinking water source, timely initiation complementary feeding, vaccination status, diarrhoea, and acute febrile illness) were candidates for the final model with p-value <0.2. However, in the final multivariable binary logistic regression model, age of the child, vitamin A supplementation for the child, weaning, and diarrhoea was significantly associated with the outcome variable (p-value<0.05).

The odds of developing acute malnutrition among female children aged 6–59 months were 1.93 times higher when compared with male children [AOR: 1.93; 95%CI (1.02, 3.65)].

The odds of developing acute malnutrition among children supplemented with vitamin A was 51% less likely as compared with those who were not supplemented with vitamin A [AOR: 0.49; 95%CI (0.25, 0.95)].

Children with not timely initiation complementary feeding were i.e., after 6 months were 2.02 times more likely to be acutely malnourished as compared with their counterparts who received complimentary food timely at 6 months of age [AOR: 2.02; 95%CI (1.06, 3.82)].

Children with diarrhoea were 2.47 times more likely to be acutely malnourished compared with those with no diarrheal episode [AOR: 2.47; 95%CI (1.28, 4.87)] (Table 2).

**Table 2:** Factors associated with acute malnutrition among 6–59 months children visiting Hamusit Health Centre from 1st September to 30th January 2021 (n = 404).

Variable	Category	Acute malnutrition		COR (95%CI)	AOR (95%CI)	p-value
		Yes (%)	No (%)			
Sex	Male	23 (10.8)	189 (89.2)	1	1	
	Female	35 (18.2)	157 (81.8)	1.83 (1.04,3.23)	1.93 (0.89, 3.65)	0.054
Age variation (months)	6–23	40 (20.8)	152 (79.2)	2.84 (1.56, 5.14)	**2.92 (1.46, 5.85)**	**0.002**
	24–59	18 (8.5)	194 (91.5)	1	1	
Family size	<5	24 (11.6)	183 (88.4)	1	1	
	≥5	34 (17.3)	163 (82.7)	1.59 (0.90, 2.79)	1.78 (0.91,3.48)	0.092
Residence	Urban	5 (7.5%)	62 (92.5)	1	1	
	Rural	53 (15.7)	284 (84.3)	2.31 (0.89,6.03)	1.63 (0.48,5.53)	0.430
Latrine availability	Yes	13 (7.6)	157 (92.4)	1	1	
	No	45 (19.2)	189 (80.8)	2.88 (1.50,5.52)	1.71 (0.74,3.98)	0.212
Vitamin A supplement	Yes	32 (10.5)	274 (89.5)	0.32 (0.18,0.58)	**0.49 (0.25,0.95)**	**0.035**
	No	26 (26.5)	72 (73.5)	1	1	
Water source	Improved	19 (9.1)	190 (90.9)	1	1	
	Un improved	39 (20.0)	156 (80.0)	2.50 (1.39, 4.50)	2.16 (0.99,4.20)	0.052
Animal and living room	separated	30 (10.5)	256 (89.5)	1	1	
	Not separated	28 (23.7)	90 (76.3)	2.66 (1.50,4.69)	1.98 (0.96,3.87)	0.056
Timely initiation complementary feeding	Yes	25 (9.8)	231 (90.2)	1	1	
	No	33 (22.3)	115 (77.7)	2.65 (1.51,4.67)	**2.02 (1.06,3.82)**	**0.032**
Vaccination status	Fully vaccinated	48 (13.2)	316 (86.6)	0.46 (0.21,0.99)	1.20 (0.48,3.03)	0.692
	Not fully vaccinated	10 (25.0)	30 (75.0)	1	1	
Diarrhoea	Yes	32 (20.3)	126 (79.7)	2.15 (1.23,3.77)	**2.47 (1.28,4.78)**	**0.007**
	No	220 (89.4)	26 (10.6)	1	1	
Acute febrile illness	Yes	36 (16.6)	181 (83.4)	1,49 (0.84, 2.64)	1.69 (0.86,3.32)	0.130
	No	22 (11.8)	165 (88.2)	1		

AOR: Adjusted Odds Ratio; COR: Crude Odds Ratio; CI: Confidence Interval; 1: Reference; Hosmer— Lemeshow Goodness- of- fit (p = 0.512).

## 4 Discussion

This cross-sectional study was conducted to assess the prevalence and associated factors of acute malnutrition among children aged 6–59 months who visited the Hamusit Health Centre. The prevalence of acute malnutrition among children aged 6–59 months who visited the Hamusit Health Centre from 1st September to 30th January was 14.4% with 95% CI (11.1%, 17.6%).

The prevalence of acute malnutrition in the hamusit health centre was higher than that of the earlier reports from Spain [32], Nepal [33], and Benishangul, Ethiopia [34]. However, it was lower than reports from Delhi, India [35], Dundee, United Kingdom [36], and Turkey [37]. The current prevalence was in line with studies from Afar, Ethiopia [38, 39], and Oromia, Ethiopia [40]. The difference in the prevalence of acute malnutrition might be due to variation in socioeconomic status, drought, agroecological difference, feeding habit, food security and availability, and season and period of the studies. Moreover, the possible justification that the present study was to be higher might be due to variation in study setting. In this regard, most of the above studies were conducted in community based while this current study was conducted in institution based (i.e., Hamusit Health Centre) which can possibly increase the prevalence of acute malnutrition. Hence, children coming to Hamusit Health Centre are potentially children who also have other underlying comorbidities. True enough, a big proportion of these children are sick.

Younger children were more acutely malnourished than older children in the present study. This was supported by studies from Nigeria [41] and South Asia [42]. However, this result was against the evidence from previous studies [43, 44, 45]. Further study on the role of age in acute malnutrition is needed to reach conclusive evidence.

It has been known that children with vitamin A supplementation were less likely to develop acute malnutrition as in the present study [46, 47, 48, 49]. Vitamin A is given both as a preventive and therapeutic measure. It is important for good vision, protecting the body against infection, and ensuring adequate growth and development [50].

Not timely initiation of complementary feeding was a risk factor for acute malnutrition among children aged 6–59 months in the present study. This may be because children above 6 months need additional food to meet nutritional requirements and late initiation of complementary feeding results in malnutrition. Initiating safe and nutritionally adequate complementary foods at an early stage (6 months) is crucial to achieve optimal growth, development, and for the health of the child [51].

Finally, children with diarrhoea preceding 2 weeks of the survey were at higher risk of acute malnutrition. This is consistent with previous results from several studies [17, 18, 19, 20, 38, 52, 53, 54]. This might be evident as diarrheal disease that affects intestinal mucosal function resulting in metabolic dysfunction, malabsorption, and nutrient loss which impaired growth, development, and nutritional status [21, 22, 23, 24].

Although this finding attempts to show the evidence gap in acute malnutrition among children in Hamusit health centre, the study inherits the following limitation. First of all, this is a single-centre study at a specific locality and hence cannot be generalized. Secondly, besides diarrheal disease, acute respiratory infection, and some covariates were assessed with caregivers’ self-report which is prone to recall and misclassification bias. Lastly, children with kwashiorkor (i.e., acute malnutrition) but their MUAC >125 mm which can underestimate the burden of acute malnutrition.

## 5 Conclusion

A significant number of children aged 6–59 months were affected by acute malnutrition. Female sex, vitamin A supplementation, not timely initiation of complementary feeding, and children with diarrhoea were factors associated with acute malnutrition.

## Declarations

### Author contribution statement

Ermias Sisay Chanie: Conceived and designed the experiments; Analyzed and interpreted the data; Wrote the paper.

Zeleke Dagne, Abenezer Melkie: Conceived and designed the experiments; Wrote the paper.

Melkamu Senbeta Jimma, Endalk Birrie Wondifraw, Fisha Alebel GebreEyesus, Getaneh Atikilt ​Yemata: Analyzed and interpreted the data; Wrote the paper.

Tahir Eyayu, Samuel Nebiyu: Performed the experiments; Analyzed and interpreted the data; Wrote the paper.

### Funding statement

This research did not receive any specific grant from funding agencies in the public, commercial, or not-for-profit sectors.

### Data availability statement

Data will be made available on request.

### Declaration of interests statement

The authors declare no conflict of interest.

### Additional information

No additional information is available for this paper.
